# 2-Chloro-4-nitro-1*H*-imidazole

**DOI:** 10.1107/S1600536810024542

**Published:** 2010-06-26

**Authors:** Hoong-Kun Fun, Jia Hao Goh, B. Chandrakantha, Arun M. Isloor, Prakash Shetty

**Affiliations:** a X-ray Crystallography Unit, School of Physics, Universiti Sains Malaysia, 11800 USM, Penang, Malaysia; b Syngene International Ltd, Biocon Park, Plot Nos. 2 & 3, Bommasandra 4th Phase, Jigani Link Road, Bangalore 560 100, India; c Organic Chemistry Division, Department of Chemistry, National Institute of Technology-Karnataka, Surathkal, Mangalore 575 025, India; d Department of Printing, Manipal Institute of Technology, Manipal University, Manipal 576 104, India

## Abstract

The mol­ecule of the title compound, C_3_H_2_ClN_3_O_2_, is almost planar; the dihedral angle between the imidazole ring and the nitro group is 1.7 (2)°. In the crystal structure, pairs of inter­molecular C—H⋯O hydrogen bonds link inversion-related mol­ecules into dimers, generating *R*_2_^2^(10) ring motifs. The dimers are inter­connected into two-dimensional networks parallel to (102) *via* inter­molecular N—H⋯N hydrogen bonds. Further stabilization is provided by short inter­molecular Cl⋯O inter­actions [3.142 (2) and 3.1475 (19) Å].

## Related literature

For general background to and applications of imidazole derivatives, see: Anuradha *et al.* (2006 ▶); Clark & Macquarrie (1996 ▶); Jadhav *et al.* (2008 ▶); Kolavi *et al.* (2006 ▶); Susanta *et al.* (2000 ▶). For graph-set descriptions of hydrogen-bond ring motifs, see: Bernstein *et al.* (1995 ▶). For related 4-nitro­imidazole crystal structures, see: Ségalas *et al.* (1992 ▶); De Bondt *et al.* (1993 ▶). For the stability of the temperature controller used for the data collection, see: Cosier & Glazer (1986 ▶).
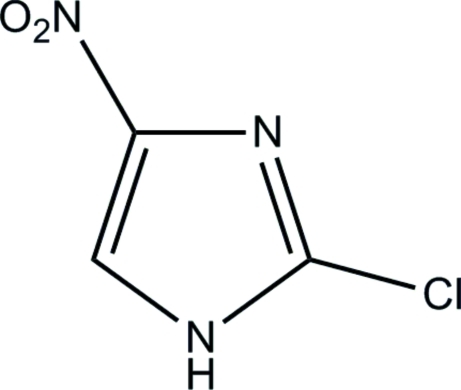


## Experimental

### 

#### Crystal data

C_3_H_2_ClN_3_O_2_
*M* *_r_* = 147.53
Monoclinic, 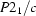

*a* = 5.905 (2) Å
*b* = 10.033 (4) Å
*c* = 9.150 (3) Å
β = 105.180 (8)°
*V* = 523.2 (3) Å^3^
*Z* = 4
Mo *K*α radiation
μ = 0.64 mm^−1^
*T* = 100 K
0.29 × 0.19 × 0.04 mm

#### Data collection

Bruker APEXII DUO CCD area-detector diffractometer
Absorption correction: multi-scan (*SADABS*; Bruker, 2009 ▶) *T*_min_ = 0.837, *T*_max_ = 0.977
5484 measured reflections
1509 independent reflections
1195 reflections with *I* > 2σ(*I*)
*R*_int_ = 0.037

#### Refinement

*R*[*F* ^2^ > 2σ(*F* ^2^)] = 0.037
*wR*(*F* ^2^) = 0.097
*S* = 1.11
1509 reflections
90 parameters
All H-atom parameters refined
Δρ_max_ = 0.42 e Å^−3^
Δρ_min_ = −0.44 e Å^−3^

### 

Data collection: *APEX2* (Bruker, 2009 ▶); cell refinement: *SAINT* (Bruker, 2009 ▶); data reduction: *SAINT*; program(s) used to solve structure: *SHELXTL* (Sheldrick, 2008 ▶); program(s) used to refine structure: *SHELXTL*; molecular graphics: *SHELXTL*; software used to prepare material for publication: *SHELXTL* and *PLATON* (Spek, 2009 ▶).

## Supplementary Material

Crystal structure: contains datablocks global, I. DOI: 10.1107/S1600536810024542/ci5106sup1.cif

Structure factors: contains datablocks I. DOI: 10.1107/S1600536810024542/ci5106Isup2.hkl

Additional supplementary materials:  crystallographic information; 3D view; checkCIF report

## Figures and Tables

**Table 1:** Hydrogen-bond geometry (Å, °)

*D*—H⋯*A*	*D*—H	H⋯*A*	*D*⋯*A*	*D*—H⋯*A*
N1—H1*N*1⋯N2^i^	0.86 (3)	2.07 (3)	2.900 (2)	163 (2)
C2—H2⋯O1^ii^	0.92 (3)	2.48 (3)	3.317 (3)	151 (2)

Symmetry codes: (i) 
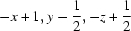
; (ii) 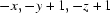.

**Fig. 1.**
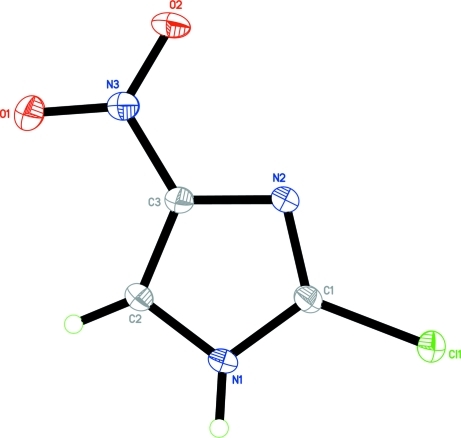
The molecular structure of the title compound, showing 50% probability displacement ellipsoids for non-H atoms and the atom-numbering scheme.

**Fig. 2.**
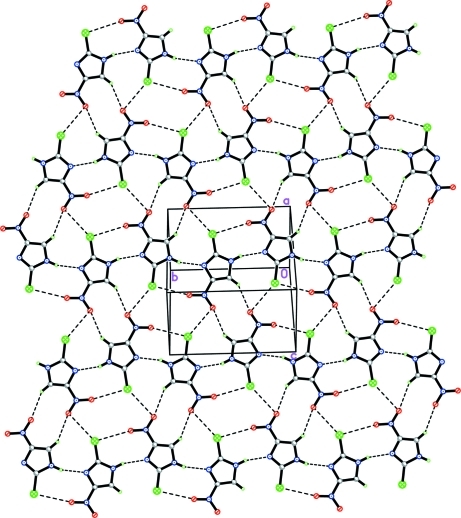
The crystal structure of the title compound, showing a two-dimensional network. Intermolecular interactions are shown as dashed lines.

## supplementary crystallographic information

### Comment

The nitro aromatic compounds are used as key substrates for the preparation of useful materials such as dyes, pharmaceuticals, perfumes and plastics (Susanta *et al.*, 2000). Therefore, nitration of hydrocarbons particularly of aromatic compounds is probably one of the most widely studied organic reactions (Jadhav *et al.*, 2008). In addition, they have proven to be valuable reagents for the synthesis of complex target molecules (Kolavi *et al.*, 2006). Most of the substituted imidazoles are widely used in pharmaceutical ingredients (Clark & Macquarrie, 1996). The imidazole nucleus is one of the important heterocyclic groups due to its presence in a large number of bioactive pharmaceutical and agrochemicals (Anuradha *et al.*, 2006). It was also reported that a large number of compounds containing the imidazole ring possess some moderately useful activities. The environmentally friendly nitration reaction has been the focus of recent research.

In the title imidazole derivative, the 1*H*-imidazole ring with atom sequence C1/N1/C2/C3/N2 is essentially planar, with a maximum deviation of 0.003 (2) Å at atom N1. The nitro group is coplanar with the attached 1*H*-imidazole ring, as indicated by the dihedral angle of 1.7 (2)°. The geometric parameters agree well with those reported for related 4-nitroimidazole structures (Ségalas *et al.*, 1992; De Bondt *et al.*, 1993).

In the crystal structure, (Fig. 2), pairs of intermolecular C2—H2···O1 hydrogen bonds (Table 1) link inversion-related molecules into dimers, generating *R*^2^_2_(10) hydrogen bond ring motifs (Bernstein *et al.*, 1995). These dimers are further interconnected into two-dimensional arrays parallel to the (102) plane *via* intermolecular N1—H1N1···N2 hydrogen bonds (Table 1). The interesting features of the crystal structure are the intermolecular short Cl···O interactions [Cl1···O1^iii^ = 3.143 (2) and Cl1···O2^i^ = 3.148 (2) Å; (i) 1-x, y-1/2, 1/2-z and (iii) 1+x, 3/2-y, z-1/2 ] which are shorter than the sum of the van der Waals radii of the relavant atoms and help to further stabilize the crystal structure.

### Experimental

Nitronium tetrafluoroborate (1.42 g, 0.0107 mol) was dissolved in nitromethane (10 ml) and 2-chloroimidazole (1 g, 0.0097 mol) was then added in lot-wise. The reaction mixture was stirred at room temperature for 3 h. The reaction mixture was then neutrallized with an aqueous solution of sodium bicarbonate. The separated solid was then filtered. The crude product was purified by column chromatography using 60–120 silica gel. The fraction eluted at 10 % ethyl acetate in hexane was concentrated to afford the title compound as pale yellow single crystals (Yield 0.9 g, 62.93 %; *m.p.* 363–366 K).

### Refinement

Atoms H1N1 and H2 were located in a difference Fourier map and allowed to refine freely [N1—H1N1 = 0.86 (3) and C2—H2A = 0.93 (3) Å].

### Crystal data

**Table:**

C_3_H_2_ClN_3_O_2_	*F*(000) = 296
*M**_r_* = 147.53	*D*_x_ = 1.873 Mg m^−^^3^
Monoclinic, *P*2_1_/*c*	Mo *K*α radiation, λ = 0.71073 Å
Hall symbol: -P 2ybc	Cell parameters from 2073 reflections
*a* = 5.905 (2) Å	θ = 3.6–30.0°
*b* = 10.033 (4) Å	µ = 0.64 mm^−^^1^
*c* = 9.150 (3) Å	*T* = 100 K
β = 105.180 (8)°	Plate, yellow
*V* = 523.2 (3) Å^3^	0.29 × 0.19 × 0.04 mm
*Z* = 4	

### Data collection

**Table:**

Bruker APEXII DUO CCD area-detector diffractometer	1509 independent reflections
Radiation source: fine-focus sealed tube	1195 reflections with *I* > 2σ(*I*)
graphite	*R*_int_ = 0.037
φ and ω scans	θ_max_ = 30.0°, θ_min_ = 3.1°
Absorption correction: multi-scan (*SADABS*; Bruker, 2009)	*h* = −6→8
*T*_min_ = 0.837, *T*_max_ = 0.977	*k* = −13→14
5484 measured reflections	*l* = −12→12

### Refinement

**Table:**

Refinement on *F*^2^	Primary atom site location: structure-invariant direct methods
Least-squares matrix: full	Secondary atom site location: difference Fourier map
*R*[*F*^2^ > 2σ(*F*^2^)] = 0.037	Hydrogen site location: inferred from neighbouring sites
*wR*(*F*^2^) = 0.097	All H-atom parameters refined
*S* = 1.11	*w* = 1/[σ^2^(*F*_o_^2^) + (0.0453*P*)^2^ + 0.1822*P*] where *P* = (*F*_o_^2^ + 2*F*_c_^2^)/3
1509 reflections	(Δ/σ)_max_ = 0.001
90 parameters	Δρ_max_ = 0.42 e Å^−^^3^
0 restraints	Δρ_min_ = −0.44 e Å^−^^3^

### Special details

**Table:**

Experimental. The crystal was placed in the cold stream of an Oxford Cryosystems Cobra open-flow nitrogen cryostat (Cosier & Glazer, 1986) operating at 100.0 (1)K.
Geometry. All esds (except the esd in the dihedral angle between two l.s. planes) are estimated using the full covariance matrix. The cell esds are taken into account individually in the estimation of esds in distances, angles and torsion angles; correlations between esds in cell parameters are only used when they are defined by crystal symmetry. An approximate (isotropic) treatment of cell esds is used for estimating esds involving l.s. planes.
Refinement. Refinement of F^2^ against ALL reflections. The weighted R-factor wR and goodness of fit S are based on F^2^, conventional R-factors R are based on F, with F set to zero for negative F^2^. The threshold expression of F^2^ > 2sigma(F^2^) is used only for calculating R-factors(gt) etc. and is not relevant to the choice of reflections for refinement. R-factors based on F^2^ are statistically about twice as large as those based on F, and R- factors based on ALL data will be even larger.

### Fractional atomic coordinates and isotropic or equivalent isotropic displacement parameters (Å^2^)

**Table:**

	*x*	*y*	*z*	*U*_iso_*/*U*_eq_	
Cl1	0.72178 (8)	0.63986 (4)	0.10842 (5)	0.01869 (15)	
O1	0.0169 (3)	0.65199 (14)	0.47907 (19)	0.0268 (4)	
O2	0.1231 (3)	0.84048 (13)	0.40048 (18)	0.0250 (3)	
N1	0.4713 (3)	0.49414 (15)	0.25596 (19)	0.0157 (3)	
N2	0.4212 (3)	0.71387 (14)	0.26450 (18)	0.0151 (3)	
N3	0.1318 (3)	0.71851 (16)	0.41138 (19)	0.0190 (3)	
C1	0.5304 (3)	0.61677 (16)	0.2149 (2)	0.0149 (4)	
C2	0.3104 (3)	0.51281 (17)	0.3371 (2)	0.0164 (4)	
C3	0.2845 (3)	0.64762 (17)	0.3405 (2)	0.0150 (4)	
H1N1	0.525 (4)	0.417 (3)	0.240 (3)	0.025 (6)*	
H2	0.246 (4)	0.441 (3)	0.375 (3)	0.025 (6)*	

### Atomic displacement parameters (Å^2^)

**Table:**

	*U*^11^	*U*^22^	*U*^33^	*U*^12^	*U*^13^	*U*^23^
Cl1	0.0218 (2)	0.0162 (2)	0.0214 (3)	−0.00093 (16)	0.01140 (19)	0.00052 (17)
O1	0.0311 (8)	0.0228 (7)	0.0340 (9)	−0.0028 (6)	0.0221 (7)	−0.0015 (6)
O2	0.0308 (8)	0.0120 (6)	0.0351 (9)	0.0043 (5)	0.0140 (7)	−0.0022 (6)
N1	0.0193 (8)	0.0096 (7)	0.0196 (8)	0.0010 (6)	0.0078 (7)	−0.0003 (6)
N2	0.0179 (8)	0.0107 (6)	0.0184 (8)	0.0001 (5)	0.0075 (6)	0.0000 (6)
N3	0.0206 (8)	0.0151 (7)	0.0231 (9)	0.0008 (6)	0.0090 (7)	−0.0017 (6)
C1	0.0174 (9)	0.0113 (8)	0.0163 (9)	−0.0013 (6)	0.0053 (7)	−0.0004 (6)
C2	0.0186 (9)	0.0114 (8)	0.0208 (10)	−0.0010 (6)	0.0082 (8)	0.0001 (7)
C3	0.0167 (9)	0.0122 (8)	0.0170 (9)	−0.0007 (6)	0.0060 (7)	−0.0019 (7)

### Geometric parameters (Å, °)

**Table:**

Cl1—C1	1.690 (2)	N2—C1	1.313 (2)
O1—N3	1.228 (2)	N2—C3	1.368 (2)
O2—N3	1.228 (2)	N3—C3	1.430 (2)
N1—C1	1.359 (2)	C2—C3	1.362 (2)
N1—C2	1.363 (3)	C2—H2	0.93 (3)
N1—H1N1	0.86 (3)		
			
C1—N1—C2	107.01 (15)	N2—C1—Cl1	124.11 (14)
C1—N1—H1N1	129.2 (17)	N1—C1—Cl1	122.87 (14)
C2—N1—H1N1	123.7 (17)	C3—C2—N1	104.32 (16)
C1—N2—C3	102.95 (15)	C3—C2—H2	135.0 (16)
O2—N3—O1	124.46 (17)	N1—C2—H2	120.7 (16)
O2—N3—C3	118.46 (16)	C2—C3—N2	112.71 (17)
O1—N3—C3	117.08 (16)	C2—C3—N3	126.29 (18)
N2—C1—N1	113.01 (17)	N2—C3—N3	120.99 (16)
			
C3—N2—C1—N1	−0.4 (2)	C1—N2—C3—C2	0.0 (2)
C3—N2—C1—Cl1	178.70 (15)	C1—N2—C3—N3	−179.05 (17)
C2—N1—C1—N2	0.6 (2)	O2—N3—C3—C2	−177.8 (2)
C2—N1—C1—Cl1	−178.52 (14)	O1—N3—C3—C2	1.9 (3)
C1—N1—C2—C3	−0.5 (2)	O2—N3—C3—N2	1.1 (3)
N1—C2—C3—N2	0.3 (2)	O1—N3—C3—N2	−179.10 (18)
N1—C2—C3—N3	179.32 (18)		

### Hydrogen-bond geometry (Å, °)

**Table:**

*D*—H···*A*	*D*—H	H···*A*	*D*···*A*	*D*—H···*A*
N1—H1N1···N2^i^	0.86 (3)	2.07 (3)	2.900 (2)	163 (2)
C2—H2···O1^ii^	0.92 (3)	2.48 (3)	3.317 (3)	151 (2)

Symmetry codes: (i) −*x*+1, *y*−1/2, −*z*+1/2; (ii) −*x*, −*y*+1, −*z*+1.

## References

[bb1] Anuradha, V., Srinivas, P. V., Aparna, P. & Madhusudana, J. R. (2006). *Tetrahedron Lett.* **47**, 4933-4935.

[bb2] Bernstein, J., Davis, R. E., Shimoni, L. & Chang, N.-L. (1995). *Angew. Chem. Int. Ed. Engl.* **34**, 1555–1573.

[bb3] Bruker (2009). *APEX2*, *SAINT* and *SADABS* Bruker AXS Inc., Madison, Wisconsin, USA.

[bb4] Clark, J. H. & Macquarrie, J. (1996). *Chem. Soc. Rev.* **25**, 3445–3446.

[bb5] Cosier, J. & Glazer, A. M. (1986). *J. Appl. Cryst.* **19**, 105–107.

[bb6] De Bondt, H. L., Ragia, E., Blaton, N. M., Peeters, O. M. & De Ranter, C. J. (1993). *Acta Cryst.* C**49**, 693–695.

[bb7] Jadhav, V. B., Kulkarni, M. V. & Rasal, V. P. (2008). *Eur. J. Med. Chem.* **43**, 1721–1729. 10.1016/j.ejmech.2007.06.023 17845827

[bb8] Kolavi, G., Hegde, V., Khan, I. z7 Gadag, P. (2006). *Bioorg. Med. Chem.* **14**, 3069–3080. 10.1016/j.bmc.2005.12.020 16406644

[bb9] Ségalas, I., Poitras, J. & Beauchamp, A. L. (1992). *Acta Cryst.* C**48**, 295–298.

[bb10] Sheldrick, G. M. (2008). *Acta Cryst.* A**64**, 112–122. 10.1107/S0108767307043930 18156677

[bb11] Spek, A. L. (2009). *Acta Cryst.* D**65**, 148–155. 10.1107/S090744490804362X PMC2631630 19171970

[bb12] Susanta, S., Frederick, F. B. & Bimal, K. B. (2000). *Tetrahedron Lett.* **41**, 8017–8020.

